# Investigation of Smartphone Addiction as a Potential Mediator of the Cross-Sectional Associations Between Body Image, Physical Activity, and Eating Disorders Among University Participants

**DOI:** 10.3390/healthcare14203541

**Published:** 2026-10-21

**Authors:** Mohammad Ahsan, Bijad S. Alqahtani

**Affiliations:** 1 Department of Physical Therapy, College of Applied Medical Sciences, Imam Abdulrahman Bin Faisal University, Dammam 31441, Saudi Arabia; 2 Department of Health Rehabilitation, College of Applied Medical Sciences at Shaqra, Shaqra University, Shaqra 11961, Saudi Arabia; balqahtani@su.edu.sa

**Keywords:** smartphone use, eating habits, physical appearance, physical activity, students

## Abstract

Background: Smartphone addiction can negatively affect university students by decreasing body satisfaction and physical activity and increasing eating disorders. However, the mechanisms underlying these detriments are unclear. This study investigates the associations among smartphone addiction, body image, physical activity, and eating disorders among university participants. Additionally, this study examines smartphone addiction as a potential mediator of these cross-sectional associations. Methods: Using a simple random sampling approach, 206 university students at Shaqra University were recruited for this cross-sectional study. Survey instruments included the Arabic version of the Smartphone Addiction Scale—Short Version (SAS-SV), the Body Image Questionnaire (BIQ-19), the International Physical Activity Questionnaire (IPAQ) (short version), and the Eating Disorder Examination Questionnaire (EDE-Q 6.0). To test mediational hypotheses, a structural equation model (SEM) was used in which smartphone addiction scores functioned as a mediator of the associations between (a) body image and eating disorder symptoms and (b) physical activity and eating disorder symptoms. Statistical significance was set at *p* < 0.05. Results: All analyses were performed in R (Version 4.4.1) using RStudio (Version: 2025.05.0+496) with the lavaan package. SEM shows that the direct effect of physical activity on eating disorder symptoms was positive and large (B = 0.64, Std. β = 0.821, SE = 0.031, 95% CI [0.576, 0.699], *p* < 0.001). This indicates that, after accounting for smartphone addiction and body image, higher physical activity is associated with higher eating disorder scores. The indirect effect of physical activity on eating disorder symptoms via smartphone addiction was positive and small but statistically significant (B = 0.02, Std. β = 0.025, SE = 0.01, 95% CI [0.004, 0.042], *p* = 0.049). This suggests that smartphone addiction partially mediates the positive relationship between physical activity and eating disorder symptoms. The total effect of physical activity on eating disorder symptoms was positive and large (B = 0.66, Std. β = 0.846, SE = 0.031, 95% CI (0.596, 0.719), *p* < 0.001). Body image did not significantly predict smartphone addiction scale scores (B = 0.022, Std. β = 0.038, SE = 0.046, 95% CI [−0.067, 0.112], *p* = 0.629), and the analysis did not provide evidence of an indirect association between body image and eating-disorder symptoms through smartphone addiction (B = −0.003, Std. β = −0.004, 95% CI [−0.015, 0.008], *p* = 0.659). Conclusions: The study identified a significant positive direct association between physical activity and eating disorder symptoms, with a small but statistically significant positive indirect effect via smartphone addiction. The total effect was positive. Body image did not significantly predict smartphone addiction scale score, and the analysis did not provide evidence of an indirect association between body image and eating-disorder symptoms through smartphone addiction. These findings are cross-sectional and do not establish temporal ordering or causal mediation. The small indirect estimate and its uncertainty should be interpreted with caution. Future research with longitudinal designs is needed to clarify the direction and mechanisms of these associations. Educational programs that incorporate smartphone use, eating habits, physical appearance, and physical activity are proposed for future evaluation rather than recommended on the basis of these findings.

## 1. Introduction

Smartphones are useful devices that have become essential to modern life, influencing various aspects of daily routines, social interactions, and health behaviors. The literature highlights both the widespread use and the potential risks associated with excessive smartphone use. It is considered a non-substance addiction, similar to internet addiction or gaming addiction, and can impact mental health, sleep, and academic or social functioning [1]. Research indicates that smartphone addiction is associated with various psychological issues such as depression, anxiety, loneliness, and sleep deprivation [2,3]. Factors like insecure attachment, fear of missing out, and poor self-control are also linked to a higher risk of developing smartphone addiction [4,5,6]. Overall, smartphone addiction is a significant concern due to its widespread prevalence among adolescents and young adults, and it warrants targeted interventions to mitigate its adverse effects.

Many studies indicate a complex relationship between smartphone use and body image. Several studies highlight that problematic smartphone behaviors, such as excessive use or smartphone addiction, are associated with increased body dissatisfaction. For instance, Xu & Shao found that higher levels of body image dissatisfaction significantly predict increased smartphone addiction, with effects varying by gender and discipline, suggesting that negative body perceptions may lead to maladaptive smartphone engagement [7]. Conversely, body dissatisfaction can also result from social comparison facilitated by social media and smartphone platforms, which may reinforce unrealistic beauty standards [8]. The evidence supports a bidirectional relationship where problematic smartphone use can exacerbate body image concerns, and pre-existing body dissatisfaction may increase vulnerability to maladaptive smartphone behaviors.

The literature suggests that smartphones may have both beneficial and adverse effects on adults’ physical activity levels. Excessive smartphone use, often characterized as smartphone addiction, has been linked to sedentary behavior and reduced physical activity, contributing to activity inequality and health risks [9]. Studies suggest that heavy smartphone use can displace time that could otherwise be spent on physical activity, thereby fostering a more sedentary lifestyle. Conversely, interventions aimed at reducing smartphone use have shown promise for increasing physical activity, indicating that limiting screen time may be an effective strategy to combat sedentary habits and promote healthier behaviors [10]. Therefore, managing smartphone use appears crucial for encouraging physical activity, especially considering the potential negative impact of smartphone addiction on movement behaviors.

Research indicates that smartphones play a significant role in both the manifestation and management of eating disorders. Many studies have explored how smartphone use, including social media and specific apps, correlates with eating disorder symptoms. For example, higher screen time and problematic social media use are associated with increased symptoms such as fear of weight gain, binge eating, and body dissatisfaction [11,12]. Additionally, internet and app usage can sometimes perpetuate disordered eating behaviors, especially when content related to dieting, beauty, or food reinforces harmful ideals [11,12,13]. Conversely, smartphones also offer promising avenues for intervention, with apps providing psychoeducation, self-monitoring, and therapy support, although their clinical utility remains under investigation [14,15]. Smartphones are a double-edged sword: they can exacerbate eating disorder symptoms but also serve as valuable tools for treatment and early detection if used appropriately.

Excessive smartphone use is associated with adverse outcomes related to body image, physical activity, and eating disorders in adolescents and young adults. Zeeni et al. [16] found that social media use correlates with body image dissatisfaction and eating disorder risk, suggesting that media content accessed via smartphones can influence perceptions of body image. Lima et al. [16,17] further demonstrated that smartphone dependence predicts poorer mental health, including increased eating disorder symptoms and lower physical activity levels, especially among younger university students. Additionally, smartphone addiction is linked to increased stress, depression, and disordered eating behaviors, which can contribute to body dissatisfaction and unhealthy eating patterns [16,17]. The evidence supports a connection between problematic smartphone use and adverse body image and eating behaviors, with reduced physical activity also being a notable concern.

Despite this growing body of literature, three critical gaps remain. First, most existing studies have examined bivariate associations between smartphone addiction and each of body image, physical activity, and eating disorders in isolation, without testing integrated models that simultaneously estimate direct and indirect pathways. Second, although bidirectionality has been documented between smartphone addiction and both body image and physical activity, the predominant theoretical framework positions behavioral addictions as proximal mechanisms through which more distal psychological and behavioral factors influence health outcomes. Specifically, smartphone addiction may serve as a behavioral means through which body dissatisfaction and sedentary behavior translate into disordered eating. Third, no study to our knowledge has examined these relationships in a Saudi Arabian university population, where cultural norms around body image, gender, and technology use may differ substantially from Western samples.

Based on the literature reviewed, the objectives of the study were to investigate the cross-sectional association among smartphone addiction, body image, physical activity, and eating disorder symptoms among university participants and to examine smartphone addiction as a potential mediator of these associations. We formulated the following pathway-specific hypotheses before analysis:

First, smartphone addiction will be positively associated with eating-disorder symptom scores. Second, physical activity will be negatively associated with eating-disorder symptom scores. Third, smartphone addiction will mediate the association between physical activity and eating-disorder symptoms. Fourth, smartphone addiction will mediate the association between body image and eating-disorder symptoms.

We emphasize that these hypotheses are theoretically motivated assumptions about the ordering of variables, not sequences established by the present cross-sectional data. This study tests indirect associations rather than establishing causal mediating mechanisms.

## 2. Methods

Study Design: A cross-sectional correlation design was selected for this study. This design is appropriate for examining associations among variables at a single time point; it does not permit causal inference.

Study settings: This study was conducted at the College of Applied Medical Sciences at Shaqra University, Shaqra, Saudi Arabia, during the 2024–2025 academic session. The College of Applied Medical Sciences includes departments of Physical Therapy, Health Rehabilitation, Nursing, and Medical Laboratory Sciences. Students from all departments were eligible to participate.

Ethical consideration: Ethical approval was granted by the Deanship of Scientific Research at Shaqra University, Shaqra, with approval number (ERC_SU_F_202300018).

Informed Consent: Prior to actual participation in the study, all participants provided informed consent, and they participated voluntarily.

Sample: A total of 206 participants (67 females [32.5%], 139 males [67.5%]) participated in this study. The mean age was 20.72 years (SD = 3.49), as mentioned in Table 1. Participants with physical or mental disabilities, cardiac problems, any chronic illness, or pregnancy were excluded because these conditions could independently affect physical activity, body image, or eating behaviors and thus confound the associations under study. The exclusion criteria were broad to ensure a relatively healthy sample.

Sampling technique: A complete list of all students enrolled in the College of Applied Medical Sciences (all four departments) was obtained from the registrar’s office. Students were selected directly from this complete college-wide list using a random number generator (https://www.random.org). This procedure constitutes simple random sampling. A total of 300 students were invited to participate; 206 completed the survey, yielding a response rate of 68.7%. No missing data were present on any of the study variables among the 206 participants included in the analysis; therefore, no listwise deletion or imputation was necessary.

Sample Size: A total of 300 students were invited to participate, and 206 completed the survey (response rate = 68.7%). The achieved sample size of 206 provided adequate precision for the estimated path coefficients, with bootstrap standard errors ranging from 0.010 to 0.054. Post hoc precision analysis indicated that the 95% confidence intervals for the direct effects were sufficiently narrow to detect moderate-to-large effects. No retrospective power calculation is reported, as it would not provide additional information beyond the confidence intervals.

Tools:

Smartphone Addiction: The Arabic version of the Smartphone Addiction Scale—Short Version (SAS-SV) was used to determine smartphone addiction among all participants. This is a self-report scale developed by Kwon et al. to assess smartphone addiction [18]. The SAS-SV consists of 10 items measured on a six-point Likert scale, ranging from 1 (completely disagree) to 6 (completely agree). The maximum score is 60, and the minimum is 10. Higher scores indicate greater smartphone addiction [18]. The original English version demonstrated excellent internal consistency, content validity, and concurrent validity [18]. Sfendla et al. evaluated the psychometric properties of the Arabic version of the SAS-SV in a Moroccan sample and found excellent reliability (Cronbach’s α = 0.87) [19]. The Arabic version was validated by El Sayed El Keshky (2022) in an Arab sample [20]. In the present sample, Cronbach’s α was 0.85. The SAS-SV classifies users into addicted (31 points or more for males and 33 points or more for females) and non-addicted (less than 31 points for males and less than 33 points for females) based on the validation study [18]. However, this classification is based on the original validation study and may not be directly applicable to our sample; therefore, we primarily used the continuous score in analyses.

Body Image: The Arabic version of the Body Image Questionnaire (BIQ-19) was used to assess body image among participants. BIQ-19 is a 19-item self-report instrument measuring individuals’ perceptions and attitudes toward their body image. This questionnaire measures multiple aspects of body satisfaction beyond simple measures of feelings, perceptions, and attitudes toward physical appearance [21]. In the Arabic version used in this study, higher total scores indicate greater body dissatisfaction (i.e., poorer body image). This is a documented difference from the original French BIQ-19, in which higher scores indicate greater satisfaction; the Arabic adaptation reversed the item polarity during translation. The BIQ-19 uses a 5-point Likert scale (1–5), yielding a total score range of 19–95. For descriptive classification, men were considered satisfied if they scored less than 48 points, while women were considered satisfied if they scored less than 45 points. These thresholds represent proportional adjustments of the original BIQ-19 cutoffs (56 for men, 53 for women) for the simple sum scoring method used in this study. BIQ-19 has been validated through extensive studies, including principal component analysis with over 1000 participants, confirming its validity and effectiveness for measuring body image across diverse populations [21]. In the present sample, Cronbach’s α was 0.82. Participants rated their body image satisfaction using the BIQ-19, providing in-depth insight into personal body image issues.

Physical activity level: The Arabic version of the International Physical Activity Questionnaire (IPAQ) (short version) was used to determine physical activity levels among college participants. The Arabic version was validated by Arumugam (2024) in an Arab sample [22]. This questionnaire was developed and validated for use by adults (15–69 years). It assesses the types and intensities of physical activity and periods of inactivity individuals engage in daily, estimating total weekly physical activity (METs) and time spent inactive. The questionnaire items are organized to provide separate scores for walking, moderate-intensity activity, and vigorous-intensity activity, as well as an overall score reflecting overall activity levels [23]. The raw MET-min/week scores (range 0–10,000) were used in all analyses. For presentation in Table 2, scores were divided by 100 (i.e., “×10^2^” indicates that the reported values are in units of 100 MET-min/week). The activity categories were defined according to the IPAQ scoring protocol: High = ≥3000 MET-min/week (or ≥1500 MET-min/week from vigorous activity); Moderate = 600–2999 MET-min/week; Low = < 600 MET-min/week. In the present sample, the mean IPAQ score was 21.14 ± 11.12 MET-min/week × 10^2^, indicating low-to-moderate physical activity levels. Cronbach’s α was 0.79.

Eating Disorder: The Arabic version of the Eating Disorder Examination Questionnaire (EDE-Q 6.0) was used to assess eating-disorder symptoms in college students. The Arabic version was validated by Aldubayan et al. (2023) in a Saudi sample [24]. EDE-Q 6.0 is a validated, 28-item self-reported tool used to measure the severity of eating disorder behaviors over the past 28 days. The questionnaire covers a variety of domains, including dietary restraint, eating concern, shape concern, and weight concern, and uses a 7-point Likert scale from 0 to 6. Subscale scores range from 0 to 6, with higher scores indicating greater eating-disorder psychopathology [25]. In the present sample, the mean EDE-Q summed subscale score was 16.63 ± 8.69 (range 0–24). The global mean score (range 0–6), calculated by dividing the sum by the number of subscales, was 4.16 ± 2.17. The summed subscale score (range 0–24) was used in all correlational and structural equation analyses. The global mean score is reported for descriptive purposes only. Cronbach’s α was 0.91. We did not classify participants as having an eating disorder, as the EDE-Q is not a diagnostic instrument and no validated caseness threshold was applied.

Statistical analysis: Descriptive statistics were calculated to summarize the study variables. Means and standard deviations were reported for continuous variables, while categorical variables were summarized using frequencies and percentages. For descriptive purposes only, some continuous variables were recoded into categories to facilitate interpretation. These categorical variables were used solely for descriptive reporting and were not entered into the structural equation model. There were no missing data on any of the study variables among the 206 participants included in the analysis; therefore, no imputation or deletion procedures were required.

To test mediational hypotheses, a structural equation model (SEM) was specified using the continuous questionnaire scores. The model was an observed-variable path model, in which smartphone addiction scores functioned as a mediator of the associations between (a) body image and eating disorder symptoms and (b) physical activity and eating disorder symptoms. The SEM included direct paths from body image and physical activity to smartphone addiction scale scores and from smartphone addiction scale scores, body image, and physical activity to eating disorder symptoms. Indirect (mediated) and total effects were defined and estimated.

The model had four observed variables and five regression paths. The model is just-identified (df = 0); therefore, global fit indices are not reported. Model parameters were estimated using maximum likelihood, and statistical inference for all effects (direct, indirect, and total) was obtained via percentile bootstrap resampling (5000 draws) to produce 95% confidence intervals (CIs) at the 2.5th and 97.5th percentiles. *p*-values for direct effects are based on bootstrap standard errors and normal approximation. *p*-values for indirect and total effects were obtained using bootstrap standard errors (5000 draws) and normal approximation. Confidence intervals are percentile bootstrap intervals for unstandardized estimates. Effects are presented as unstandardized coefficients (B), standardized coefficients (Std. β), standard errors (SEs), 95% bootstrap CIs, and two-tailed *p*-values. Standardized coefficients were computed using the observed variances of the endogenous and exogenous variables. Statistical significance was set at *p* < 0.05. All analyses were performed in R (Version 4.4.1) using RStudio (Version: 2025.05.0+496). The structural equation model was fitted using the lavaan package (Version 0.6-19) for R. Bootstrap confidence intervals were computed using the boot package (Version 1.3-30).

## 3. Results

To analyze the data for this study, continuous scores were used. Categorical classifications are presented for descriptive purposes only. The demographic profile of participants is presented in Table 1. The study included 206 university participants with a mean age of 20.72 years (SD = 3.49). The average height and weight were 168.13 cm (SD = 11.10) and 70.71 kg (SD = 21.52), respectively. There were 67 females (32.5%) and 139 males (67.5%).

Table 2 reports mean values of 34.13 ± 8.41 for smartphone addiction, 80.12 ± 4.14 for body image, 21.14 ± 11.12 for physical activity, and 16.63 ± 8.69 for eating disorder.

Table 3 presents stratified descriptive statistics for each variable. For categorical indicators, just over half the sample were classified as addicted on the smartphone addiction scale measure (106/206, 51.5%). Non-addicted cases numbered 100 (48.5%). Regarding body image, 100 participants (48.5%) were classified as satisfied, and 106 (51.5%) were classified as unsatisfied. Physical activity levels were distributed as follows: High = 46 (22.3%), Moderate = 109 (52.9%), and Low = 51 (24.8%). Eating-disorder symptoms were analyzed as a continuous variable (EDE-Q summed subscale score: mean = 16.63, SD = 8.69, range 0–24). No categorical classification was applied.

Table 4 shows that smartphone addiction negatively correlates with physical activity (r = −0.252, *p* < 0.001) and eating disorder symptoms (r = −0.314, *p* < 0.001), indicating that higher smartphone addiction is associated with lower physical activity and lower eating-disorder symptom scores. The relationship between smartphone addiction and body image was negligible and nonsignificant (r = 0.021, *p* = 0.760). Body image was inversely related to physical activity (r = −0.374, *p* < 0.001) and eating disorder symptoms (r = −0.302, *p* < 0.001), suggesting that poorer body image (higher BIQ-19 scores) is associated with reduced physical activity and lower eating-disorder symptom scores. The correlation between physical activity and eating disorder symptoms is r = 0.780, *p* < 0.001, indicating a strong positive relationship. This means that higher levels of physical activity are associated with higher eating disorder symptom scores (i.e., more eating disorder symptoms).

**Figure 1:**
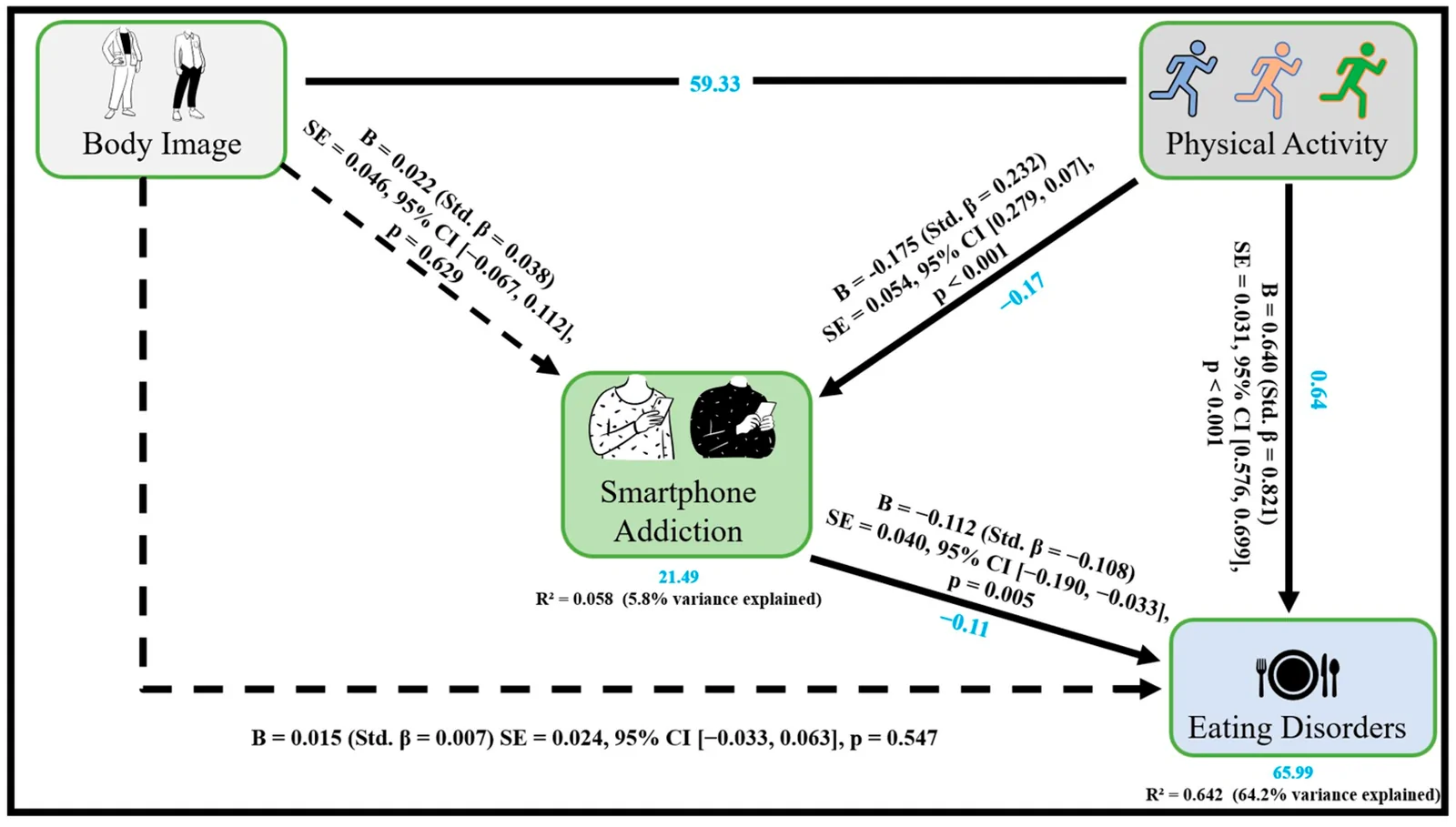
Structural equation model for smartphone addiction, body image, physical activity, and eating disorder. The figure shows a path diagram with four observed variables: Body Image (BIQ-19), Physical Activity (IPAQ-SF), Smartphone Addiction (SAS-SV), and Eating Disorder (EDE-Q 6.0). Single-headed arrows represent regression paths. Solid arrows indicate significant paths; dashed arrows indicate nonsignificant paths. Path coefficients are labeled with B values and standardized β values in parentheses. R^2^ = 0.058 for Smartphone Addiction; R^2^ = 0.642 for Eating Disorder. Residual variances: Smartphone Addiction = 21.49; Eating Disorder = 65.99. Structural equation model testing smartphone addiction scale scores as a mediator between body image, physical activity, and eating disorder symptoms. Solid lines = significant paths (*p* < 0.05); dashed lines = nonsignificant paths (*p* > 0.05). Values are unstandardized coefficients (B) with standardized coefficients (Std. β) in parentheses. R^2^ values are shown for endogenous variables. Residual variances are shown in green for endogenous variables. N = 206.

SEM Model results are presented in Table 5 and Figure 1. The direct effect of physical activity on eating disorder symptoms is B = 0.64, Std. β = 0.821, SE = 0.031, 95% CI [0.576, 0.699], *p* < 0.001, which is also positive and large. This indicates that, after accounting for smartphone addiction scale score and body image, higher physical activity is associated with higher eating disorder symptom scores. The indirect effect of physical activity on eating disorder symptoms via smartphone addiction is B = 0.02, Std. β = 0.025, SE = 0.01, 95% CI [0.004, 0.042], *p* = 0.049, which is positive and small but significant. This suggests that smartphone addiction partially mediates the positive relationship between physical activity and eating disorder symptoms. The total effect of physical activity on eating disorders is B = 0.66, Std. β = 0.846, SE = 0.031, 95% CI (0.596, 0.719), *p* < 0.001, which is positive and large.

Predictors of the mediator (Smartphone Addiction Scale Score): Physical activity level significantly predicted smartphone addiction scale score in the expected direction: higher physical activity level was associated with lower smartphone addiction scale score (B = −0.175, Std. β = −0.232, SE = 0.054, 95% CI [−0.279, −0.070], *p* = 0.001). Body image did not significantly predict smartphone addiction scale score (B = 0.022, Std. β = 0.038, SE = 0.046, 95% CI [−0.067, 0.112], *p* = 0.629).

Indirect and total effects: The indirect effect of physical activity on eating disorder symptoms, operating via the smartphone addiction scale score, was small but statistically significant (indirect B = 0.02, Std. β = 0.025, SE = 0.01, 95% CI [0.004, 0.042], *p* = 0.049). However, the direct effect was much larger (Std. β = 0.821), indicating that smartphone addiction serves as a weak partial mediator. By contrast, the indirect effect of body image on eating disorder symptom scores via smartphone addiction scale score was essentially zero and not significant (indirect B = −0.003, Std. β = −0.004, 95% CI [−0.015, 0.008], *p* = 0.659), indicating that the analysis did not provide evidence of an indirect association. The total effect of physical activity on eating disorder symptoms (direct + indirect) remained large and significant (total B = 0.66, Std. β = 0.846, SE = 0.031, 95% CI [0.596, 0.719], *p* < 0.001). The total effect of body image on eating disorder symptoms was not significant (total B = 0.012, Std. β = 0.020, *p* = 0.608).

The model accounted for a modest proportion of variance in the mediator (R^2^ for smartphone addiction scale score = 0.058) and a substantial proportion of variance in the outcome (R^2^ for eating disorder symptoms = 0.642), as shown in Figure 1.

## 4. Discussion

The purpose of this study was to investigate the mediating effects of smartphone addiction on body image, physical activity, and eating disorders in university participants. The findings revealed that the prevalence of smartphone addiction was 51.5%. 51.5% of participants were not satisfied with their body image. Notably, 52.9% of participants were moderately physically active. The mean EDE-Q continuous variable score was 16.63 ± 8.69. Furthermore, the smartphone addiction scale score had a small but statistically significant negative direct association with eating disorder symptoms. Overall, these findings highlight smartphone addiction’s consistent association with key psychological and behavioral outcomes, especially eating-disorder symptoms and physical activity. Body image did not have a significant direct effect on eating-disorder scores. Physical activity level significantly predicted smartphone addiction scale score in the expected direction: higher physical activity level was associated with lower smartphone addiction scale score. Body image did not significantly predict the smartphone addiction scale score.

We note that the direction of some associations was unexpected. Specifically, smartphone addiction was negatively associated with eating-disorder symptoms, whereas physical activity was positively associated with eating-disorder symptoms. This contrasts with much of the existing literature, which generally reports positive associations between smartphone addiction and eating-disorder symptoms and negative associations between physical activity and eating-disorder symptoms. Several explanations are possible, and we present them here as hypotheses for future testing rather than conclusions established by these data. First, the EDE-Q measures a range of eating-disorder psychopathology, and it is possible that in this sample, individuals with higher smartphone addiction scores reported lower EDE-Q scores due to differences in reporting or interpretation of items. Second, the positive association between physical activity and eating-disorder symptoms may reflect that individuals with eating-disorder symptoms engage in compensatory or excessive exercise, which is a common feature of eating disorders; however, this study did not measure exercise motivation or compulsivity, so this explanation remains hypothetical. Third, the cross-sectional design precludes causal interpretation, and reverse causality or confounding may explain these findings. Future research with longitudinal designs and objective measures is needed to clarify these associations.

Smartphone addiction, as evidenced in the literature, is associated with a range of negative psychological and behavioral outcomes in young adults. It is linked to increased body dissatisfaction, disordered eating behaviors, and mental health issues such as depression, anxiety, and stress [17,26]. Several studies have found that higher levels of smartphone dependency correlate with body image distortion, particularly among adolescents and university students [17,27]. Mechanistically, smartphone addiction appears to mediate the relationship between body dissatisfaction and disordered eating, possibly through emotional regulation difficulties and mental health impairments [26]. Multiple studies suggest that higher levels of problematic smartphone use are associated with greater body dissatisfaction, particularly among young adults and college students [7,28]. This relationship appears to be mediated by psychological factors such as intrusive imagery, fear of negative evaluation, and difficulties with emotion regulation [7,28]. Furthermore, body dissatisfaction may be a risk factor for problematic smartphone behaviors, especially in contexts where social media and image-focused content are prevalent [7]. These findings suggest that smartphone addiction may serve as a correlate of body image concerns and related psychopathologies, emphasizing the importance of addressing problematic smartphone use in interventions targeting body image and eating disorder symptoms. However, our study did not find a significant association between body image and smartphone addiction, which may be due to the specific characteristics of our sample or the measures used.

The findings of our study are consistent with the findings of many researchers. Studies indicate that individuals with higher levels of smartphone addiction tend to have lower levels of physical activity and are more likely to engage in sedentary behaviors [17,26]. For instance, Lima et al. found that smartphone addiction was associated with reduced physical exercise, and this pattern was associated with poorer mental health outcomes [17]. Similarly, Yağci Şentürk et al. observed that smartphone addiction correlated with sedentary lifestyles and neck disability, which could further limit physical activity [29]. It seems that smartphone addiction appears to be a significant factor contributing to decreased physical activity and increased sedentary behavior. Nikolic et al. found that excessive smartphone use among medical students was associated with poorer sleep quality and higher levels of stress, anxiety, and depression, which can negatively impact motivation and capacity for physical activity [30]. Similarly, Yaşarer et al. suggest that heavy smartphone use may lead to physical discomfort and reduced engagement in physical activity [31]. Furthermore, Putchavayala et al. highlight that yoga, a form of physical activity, is proposed as a holistic intervention to address smartphone addiction, emphasizing its role in self-regulation and de-addiction processes [32]. Kim underscores exercise rehabilitation as a promising approach to treat smartphone addiction, indicating that physical activity can mitigate physical and mental symptoms associated with excessive smartphone use [33]. Overall, the evidence supports the view that increased physical activity can serve as a protective factor against smartphone addiction, and interventions that promote physical activity may help reduce problematic smartphone use. However, our cross-sectional design cannot establish temporal precedence.

The literature indicates a significant association between smartphone addiction and eating disorders, particularly among young adults and university students. Multiple studies demonstrate that higher levels of smartphone addiction correlate positively with disordered eating behaviors, body dissatisfaction, and food addiction [28,34,35]. These relationships are mediated by psychological factors such as depression, anxiety, and emotional regulation difficulties, suggesting that smartphone addiction may contribute to eating disorder symptoms through these mental health pathways [26,36]. Additionally, problematic smartphone use and increased screen time are linked to a higher prevalence of binge eating, purging behaviors, and higher BMI, further supporting the connection between digital media behaviors and eating disorder pathology [34]. Overall, the evidence supports a model where smartphone addiction acts as a correlate of disordered eating, mediated by mental health disturbances, although most studies are cross-sectional, limiting causal inferences [37]. Furthermore, several studies, including Wang et al. and Yılmaz et al., demonstrate that higher levels of smartphone addiction correlate positively with disordered eating behaviors, such as restrictive, emotional, and external eating [38,39]. These associations are mediated by factors like body dissatisfaction, depression, and stress, suggesting that smartphone addiction may contribute to eating disorder symptoms through psychological pathways. Furthermore, research by Yang et al. highlights that smartphone addiction can influence body image dissatisfaction and disordered eating via mediators like depression, reinforcing the interconnectedness of these issues [26]. Overall, the evidence supports a close link between smartphone addiction and eating disorders, emphasizing the importance of addressing problematic smartphone use in interventions aimed at reducing disordered eating behaviors. Our finding of a negative association between smartphone addiction and eating-disorder symptoms is contrary to much of this literature and may reflect sample-specific characteristics or measurement issues.

Contrary to our hypothesis and much of the existing literature, we found a negative direct association between smartphone addiction and eating disorder symptoms in the SEM. The association between smartphone addiction and eating-disorder symptoms changed direction after adjustment for physical activity, which may reflect the shared variance between these variables. However, this study cannot determine the reason for this change. Individuals with high smartphone addiction may be more sedentary and less likely to engage in maladaptive exercise, which is a key behavioral symptom of eating disorders; however, this study did not measure exercise motivation or compulsivity, so this explanation remains hypothetical. Additionally, cultural and sample-specific factors may play a role, as our sample consists of Saudi university students, who may differ from populations in previous studies. Future research should explore these possibilities using longitudinal designs and objective measures of physical activity and eating disorder behaviors.

Contrary to our hypothesis and much of the existing literature, body image did not significantly predict smartphone addiction or eating disorder symptoms in our sample. This null finding may reflect measurement limitations of the BIQ-19, which measures general body image satisfaction rather than appearance-related concerns specific to social media or eating disorders. Additionally, cultural factors specific to Saudi university students may influence these relationships. It is also possible that suppression effects, due to the inclusion of physical activity in the model, attenuated the body image effects. Future research should use multidimensional measures of body image and explore these relationships in diverse cultural contexts.

Body image and physical activity are interconnected factors that influence eating behaviors and the development or maintenance of eating disorders. Engaging in physical activity can positively impact body image by promoting feelings of competence, strength, and well-being, which may enhance self-esteem and reduce body dissatisfaction. However, in the context of eating disorders, physical activity often becomes maladaptive, characterized by compulsive or excessive exercise aimed at controlling weight or shape, which can reinforce negative body image and perpetuate disordered eating behaviors [40]. The literature emphasizes the importance of distinguishing between adaptive and maladaptive exercise, as the latter is associated with adverse mental and physical health outcomes and can hinder recovery. Therefore, while physical activity has potential benefits for body image, its role in eating disorder treatment must be carefully managed to prevent the reinforcement of harmful behaviors [40]. Many studies show a strong association between body image and eating disorders, with negative body image serving as both a risk factor and a consequence of disordered eating behaviors. Adolescents and young adults are particularly vulnerable, as societal and media influences promote idealized body standards that often lead to body dissatisfaction [41,42]. Negative perceptions of body image can contribute to unhealthy weight control practices, restrictive dieting, and other disordered eating patterns, which may evolve into clinical eating disorders such as anorexia nervosa or bulimia nervosa [41]. Conversely, individuals with eating disorders frequently report distorted body image perceptions, emphasizing the bidirectional relationship. Interventions targeting body image improvement have shown promise in reducing disordered eating behaviors, underscoring the importance of addressing body image issues within prevention and treatment frameworks [43]. Overall, the literature underscores that body image disturbances are central to the development and maintenance of eating disorders, necessitating integrated approaches that focus on both psychological and behavioral components.

Limitations: While this investigation offers valuable insights into the associations and potential mediating effects of smartphone addiction on body image, physical activity, and eating-disorder symptoms, it is important to interpret the findings with caution due to certain limitations. First, the cross-sectional design precludes causal inference and does not allow for temporal ordering of variables; the indirect effects reported here represent cross-sectional associations, not causal mediation. Second, the self-report method was used to measure smartphone addiction, body image, physical activity, and eating-disorder symptoms, which may introduce common-method bias and social desirability bias. Future research could benefit from more objective data collection methods. Third, recruitment from a single university and over a short window limits generalizability to other settings. Fourth, the sex imbalance (32.5% female, 67.5% male) may limit generalizability to female-dominated populations. Fifth, although key sociodemographic characteristics were recorded, unmeasured confounding (e.g., habitual physical activity, dietary details, mental health symptoms) may remain. Sixth, potential selection bias may have occurred if students who chose to participate differed systematically from those who did not. Seventh, the EDE-Q is not a diagnostic instrument, and we did not apply a validated caseness threshold; therefore, we cannot report the prevalence of eating disorders. Eighth, the physical activity measure (IPAQ short version) may be subject to recall bias and may not capture all domains of activity. Ninth, the sample size, while adequate for the SEM, may have limited power to detect small effects. Tenth, the BIQ-19 classification thresholds were proportionally adjusted for the Arabic version’s scoring direction and simple sum scoring method, which may affect comparability with studies using the original weighted scoring algorithm. Finally, the model is just-identified (df = 0), so global fit indices cannot be reported, and the model cannot be rejected or confirmed on the basis of fit.

## 5. Conclusions

This study identified a significant positive direct association between physical activity and eating disorder symptoms, with a small but statistically significant positive indirect effect via smartphone addiction. The total effect of physical activity on eating disorder symptoms was positive and large. Body image did not significantly predict smartphone addiction scale score, and the analysis did not provide evidence of an indirect association between body image and eating disorder symptoms through smartphone addiction. These findings are cross-sectional and do not establish temporal ordering or causal mediation. The small indirect estimate and its uncertainty should be interpreted with caution. Future research with longitudinal designs is needed to clarify the direction and mechanisms of these associations. Educational programs that incorporate smartphone use, eating habits, physical appearance, and physical activity are proposed for future evaluation rather than recommended on the basis of these findings.

## Figures and Tables

**Table 1:** Demographic characteristics.

	Overall
	(N = 206)
**Age (in Years)**	
Mean ± SD	20.72 ± 3.49
**Height (cm)**	
Mean ± SD	168.13 ± 11.10
**Weight (Kg)**	
Mean ± SD	70.71 ± 21.52
**Gender [n(%)]**	
Female	67 (32.5%)
Male	139 (67.5%)

**Table 2:** Descriptive statistics of scales.

Variables	Mean ± SD	Range
**Smartphone Addiction**		
(SAS-SV score)	34.13 ± 8.41	10–16
**Body Image**		
(BIQ-19 score)	80.12 ± 4.14	19–95
**Physical Activity**		
MET-min/week × 10^2^	21.14 ± 11.12	0–100
**Eating disorder**		
(EDE-Q sum Score)	16.63 ± 8.69	0–24

Note: Physical activity scores are presented as MET-min/week × 10^2^ (i.e., divided by 100 for presentation). The raw MET-min/week scores (range 0–10,000) were used in all analyses. EDE-Q global mean score = 4.16, SD = 2.17, range 0–6. The summed EDE-Q subscale score (range 0–24) was used in all correlational and structural equation analyses; the global mean score is reported for descriptive purposes only.

**Table 3:** Descriptive statistics (frequency distribution) of each subscale.

Variables (N = 206)	n (%)
**Smartphone Addiction**	
Addicted	106 (51.5%)
Non-addicted	100 (48.5%)
**Body Image**	
Satisfied	100 (48.5%)
Un-satisfied	106 (51.5%)
**Physical Activity**	
High	46 (22.3%)
Moderate	109 (52.9%)
Low	51 (24.8%)
**Eating disorder** *	
Continuous variable	Mean = 16.63, SD = 8.69

* Eating-disorder symptoms were analyzed as a continuous variable (EDE-Q summed subscale score, range 0–24). No categorical classification was applied, as the EDE-Q is not a diagnostic instrument and no validated caseness threshold was applied.

**Table 4:** Relationship among smartphone addiction, body image, physical activity, and eating disorders.

		Smartphone Addiction	Body Image	Physical Activity
**Smartphone Addiction**		1		
**Body Image**	r	0.021	1	
	p	(0.760)		
**Physical Activity**	r	−0.252	−0.374	1
	p	(<0.001)	(<0.001)	
**Eating Disorder**	r	−0.314	−0.302	0.780
	p	(<0.001)	(<0.001)	(<0.001)

**Table 5:** SEM parameter estimates (bootstrap CIs).

Type	Parameter	B	Std. β	SE	95% CI	*p*
Direct	Eating Disorder–Body Image	0.015	0.007	0.024	(−0.033, 0.063)	0.547
Direct	Eating Disorder–Physical Activity	0.640	0.821	0.031	(0.576, 0.699)	<0.001
Direct	Eating Disorder–Smartphone Addiction	−0.112	−0.108	0.040	(−0.190, −0.033)	0.005
Direct	Smartphone Addiction–Body Image	0.022	0.038	0.046	(−0.067, 0.112)	0.629
Direct	Smartphone Addiction–Physical Activity	−0.175	−0.232	0.054	(−0.279, −0.070)	0.001
Indirect/Total	Indirect Physical Activity	0.020	0.025	0.010	(0.004, 0.042)	0.049
Indirect/Total	Indirect Body Image	−0.003	−0.004	0.006	(−0.015, 0.008)	0.659
Indirect/Total	Total Physical Activity	0.660	0.846	0.031	(0.596, 0.719)	<0.001
Indirect/Total	Total Body Image	0.012	0.020	0.024	(−0.033, 0.060)	0.608

The model was an observed-variable path model with four observed variables and five regression paths. The model is just-identified (df = 0); therefore, global fit indices are not reported. Standardized coefficients were computed using the observed variances of the endogenous and exogenous variables.

## Data Availability

The raw data supporting the conclusions of this article will be made available by the authors on request.
